# The genome sequence of the ruby bryozoan, *Bugula neritina *(Linnaeus, 1758)

**DOI:** 10.12688/wellcomeopenres.23056.1

**Published:** 2024-09-18

**Authors:** Rebekka Uhl, John Bishop, Helen Jenkins, Christine Wood, Patrick Adkins, Freja Azzopardi, Marine Biological Association Genome Acquisition Lab, Darwin Tree of Life Barcoding collective, Wellcome Sanger Institute Tree of Life Management, Samples and Laboratory team, Wellcome Sanger Institute Scientific Operations: Sequencing Operations, Wellcome Sanger Institute Tree of Life Core Informatics team, Tree of Life Core Informatics collective, Darwin Tree of Life Consortium

**Affiliations:** 1 The Marine Biological Association, Plymouth, England, UK

**Keywords:** Bugula neritina, ruby bryozoan, genome sequence, chromosomal, Cheilostomatida

## Abstract

We present a genome assembly from a specimen of *Bugula neritina* (the ruby bryozoan; Bryozoa; Gymnolaemata; Cheilostomatida; Bugulidae). The genome sequence has total length of 216.00 megabases. Most of the assembly is scaffolded into 9 chromosomal pseudomolecules. The mitochondrial genome has also been assembled and is 15.25 kilobases in length. Gene annotation of this assembly on Ensembl identified 20,264 protein-coding genes.

## Species taxonomy

Eukaryota; Opisthokonta; Metazoa; Eumetazoa; Bilateria; Protostomia; Spiralia; Lophotrochozoa; Bryozoa; Gymnolaemata; Cheilostomatida; Flustrina; Buguloidea; Bugulidae; Bugula; *Bugula neritina* (Linnaeus, 1758) (NCBI:txid10212).

## Background

The erect colonies of the bryozoan *Bugula neritina* are commonly deep purplish-brown, and are formed of narrow, dichotomously dividing branches, two zooids wide ( Bilewitch, 2009; Hayward & Ryland, 1995). They could be mistaken for finely branched red seaweeds, but are much tougher in texture. The species was first described from the Mediterranean Sea. However, records of the bryozoan are widespread, ranging from New Zealand, the Atlantic and Gulf coast of America and Hawai’i. *B. neritina* is a highly successful fouling organism and is now also commonly found in the UK as an invasive species, especially within fouling communities ( Bilewitch, 2009; McGovern & Hellberg, 2003). 

Molecular research has shown that *B. neritina* is in fact a complex of at least three cryptic species, Types S, D and N ( Davidson & Haygood, 1999). In addition to molecular characteristics, these species vary by habitat and geographical location. Type N (North Atlantic) and D (Deep) are mostly restricted to North America, whereas type S (Shallow) is widely distributed globally and is commonly found in the UK ( Fehlauer-Ale *et al.*, 2014).

*B. neritina* was once thought to be the producer of bryostatins (cancer-fighting cyclic polyketides), but it has since been demonstrated that a bacterial symbiont *Candidatus* Endobugula sertula is responsible for bryostatin production ( Davidson *et al.*, 2001). The production of these bryostatins is of ecological importance for the bryozoan as they are used to coat the larvae for protection ( Sharp *et al.*, 2007). The presence of bryostatins in the coat has been found to be unpalatable to predators and thereby allows the larvae to settle successfully despite their large size and nutritional value ( Lindquist & Hay, 1996; McGovern & Hellberg, 2003). Interestingly, despite the importance of this bacterial symbiosis, type N *B. neritina* has only been seen to exhibit the symbiont when it is found further south of its usual distribution ( Linneman *et al.*, 2014). This may be a consequence of lower predation pressure in the Northern range of Type N distribution; hence bryostatin production is less ecologically important ( McGovern & Hellberg, 2003).

Molecular research into these organisms will further provide an understanding of host-symbiont interactions, for example, by analysing the presence or absence of genes that control bryostatin production ( Hildebrand *et al.*, 2004; Miller *et al.*, 2016). A draft genome of *B. neritina* has previously been published (GCA_010799875.2) and used to better understand the evolution of bryozoans, for example via the expression of the Hox gene cluster ( Rayko *et al.*, 2020; Saadi *et al.*, 2023). Thus, the publication of the complete genome sequence of *B. neritina* expected to have a significant impact, particularly due to the pharmacological importance of bryostatins.

## Genome sequence report

**Figure 1.**
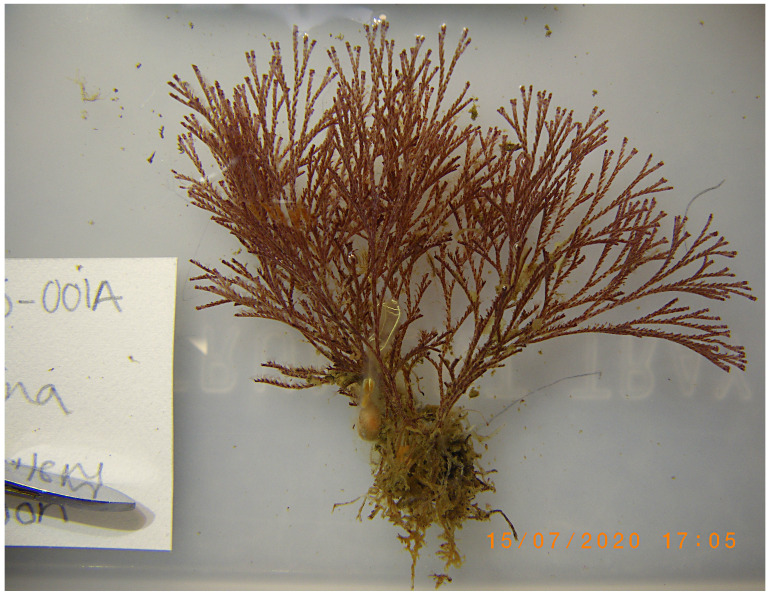
Photograph of the *Bugula neritina* (tzBugNeri2) specimen used for genome sequencing.

The genome of an adult *Bugula neritina* ( Figure 1) was sequenced using Pacific Biosciences single-molecule HiFi long reads, generating a total of 27.37 Gb (gigabases) from 2.82 million reads, providing approximately 132-fold coverage. Primary assembly contigs were scaffolded with chromosome conformation Hi-C data, which produced 85.23 Gbp from 564.45 million reads, yielding an approximate coverage of 395-fold. Specimen and sequencing information is summarised in Table 1.

**Table 1.** Specimen and sequencing data for *Bugula neritina*.

Project information			
**Study title**	*Bugula neritina* (ruby bryozoan)		
**Umbrella BioProject**	PRJEB66388		
**Species**	*Bugula neritina*		
**BioSample**	SAMEA112148689		
**NCBI taxonomy ID**	10212		
Specimen information			
**Technology**	**ToLID**	**BioSample accession**	**Organism part**
**PacBio long read sequencing**	tzBugNeri2	SAMEA112152753	Modular colony
**Hi-C sequencing**	tzBugNeri1	SAMEA7536669	Modular colony
Sequencing information			
**Platform**	**Run accession**	**Read count**	**Base count (Gb)**
**Hi-C Illumina NovaSeq 6000**	ERR12102386	5.64e+08	85.23
**PacBio Sequel IIe**	ERR12085103	2.82e+06	27.37

**Figure 2.**
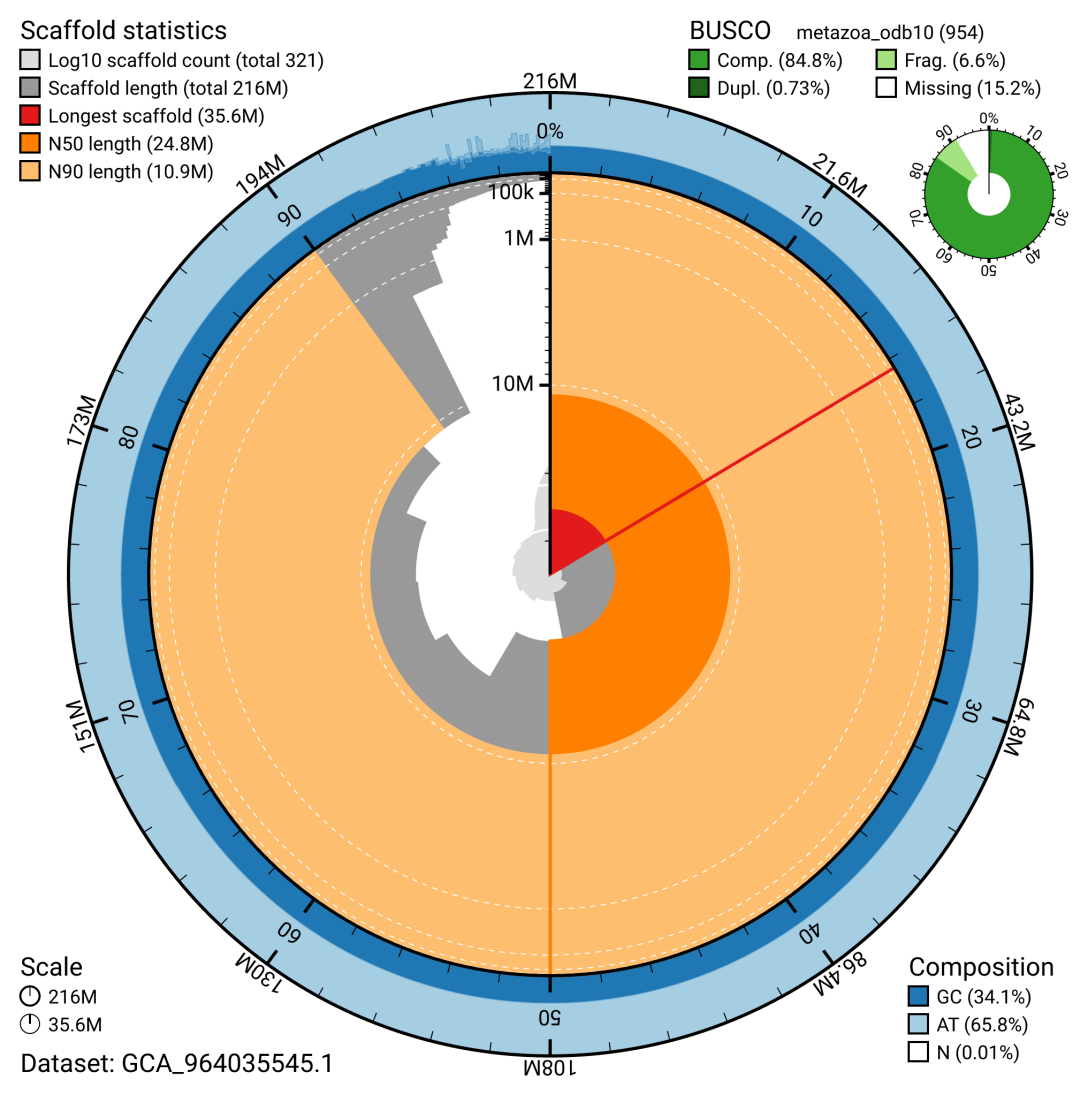
Genome assembly of *Bugula neritina*, tzBugNeri2.1: metrics. The BlobToolKit snail plot shows N50 metrics and BUSCO gene completeness. The main plot is divided into 1,000 size-ordered bins around the circumference with each bin representing 0.1% of the 216,009,554 bp assembly. The distribution of scaffold lengths is shown in dark grey with the plot radius scaled to the longest scaffold present in the assembly (35,640,107 bp, shown in red). Orange and pale-orange arcs show the N50 and N90 scaffold lengths (24,795,343 and 10,879,868 bp), respectively. The pale grey spiral shows the cumulative scaffold count on a log scale with white scale lines showing successive orders of magnitude. The blue and pale-blue area around the outside of the plot shows the distribution of GC, AT and N percentages in the same bins as the inner plot. A summary of complete, fragmented, duplicated and missing BUSCO genes in the metazoa_odb10 set is shown in the top right. An interactive version of this figure is available at https://blobtoolkit.genomehubs.org/view/GCA_964035545.1/dataset/GCA_964035545.1/snail.

**Figure 3.**
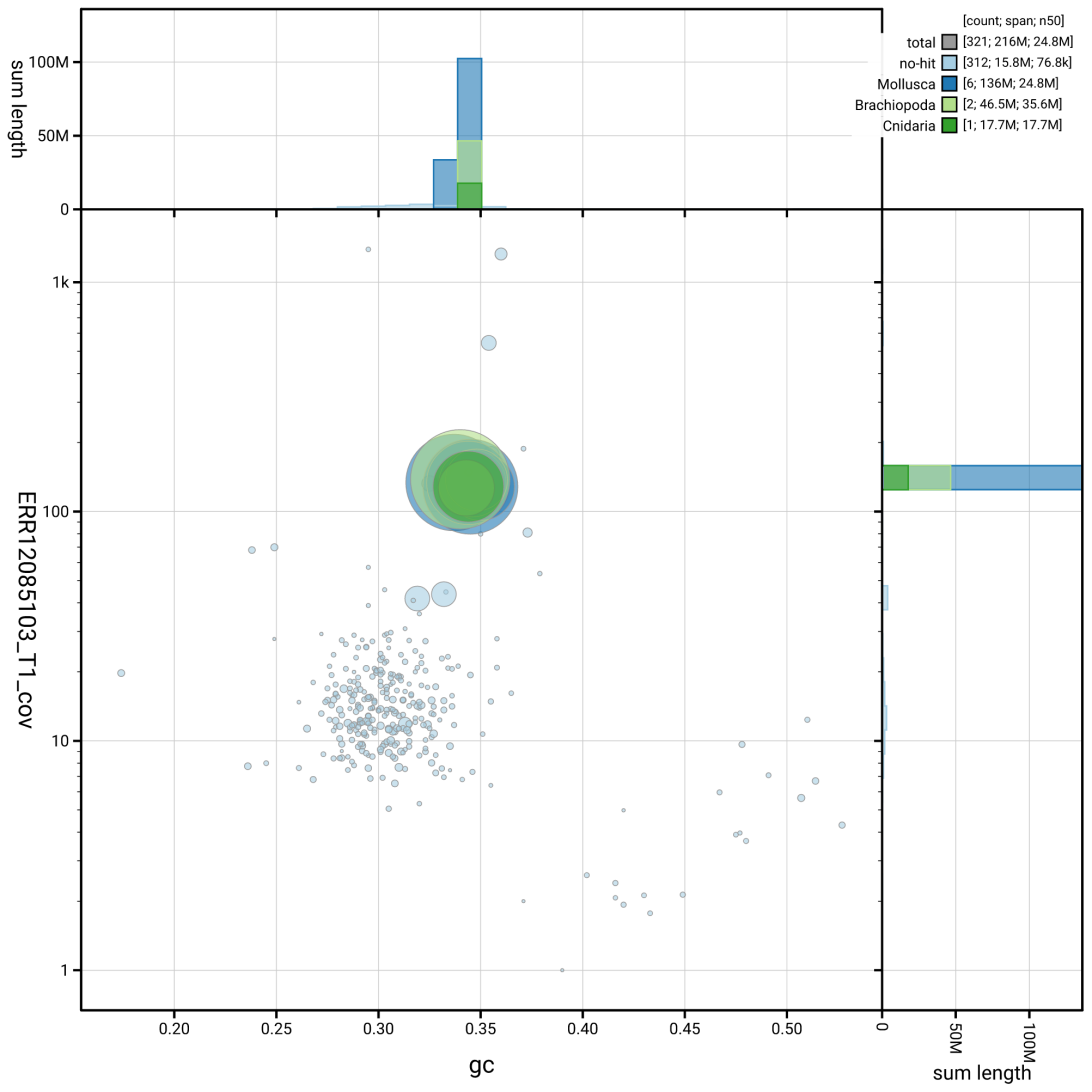
Genome assembly of *Bugula neritina*, tzBugNeri2.1: BlobToolKit GC-coverage plot. Sequences are coloured by phylum. Circles are sized in proportion to sequence length. Histograms show the distribution of sequence length sum along each axis. An interactive version of this figure is available at https://blobtoolkit.genomehubs.org/view/GCA_964035545.1/dataset/GCA_964035545.1/blob.

**Figure 4.**
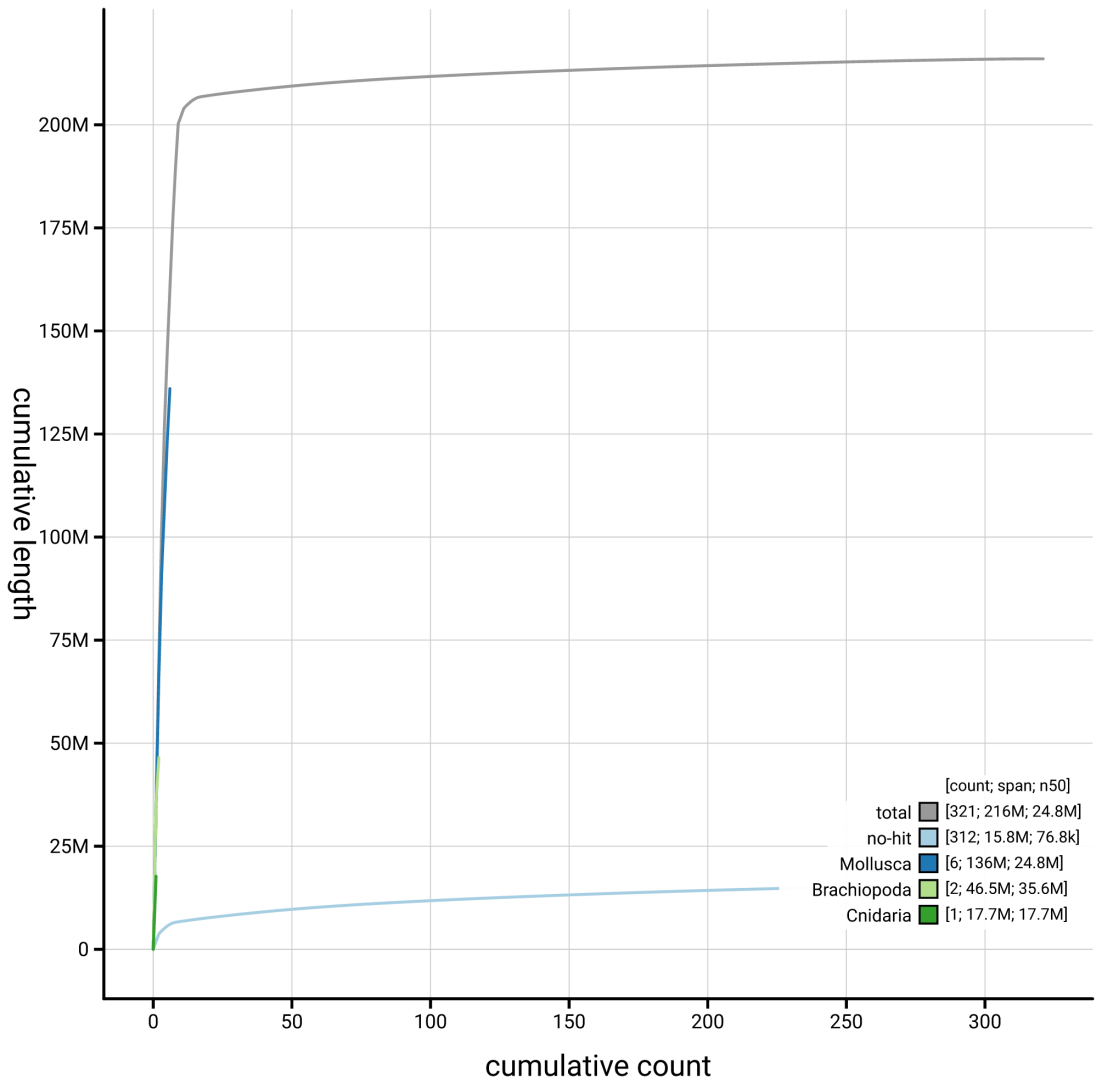
Genome assembly of *Bugula neritina* tzBugNeri2.1: BlobToolKit cumulative sequence plot. The grey line shows cumulative length for all sequences. Coloured lines show cumulative lengths of sequences assigned to each phylum using the buscogenes taxrule. An interactive version of this figure is available at https://blobtoolkit.genomehubs.org/view/GCA_964035545.1/dataset/GCA_964035545.1/cumulative.

**Figure 5.**
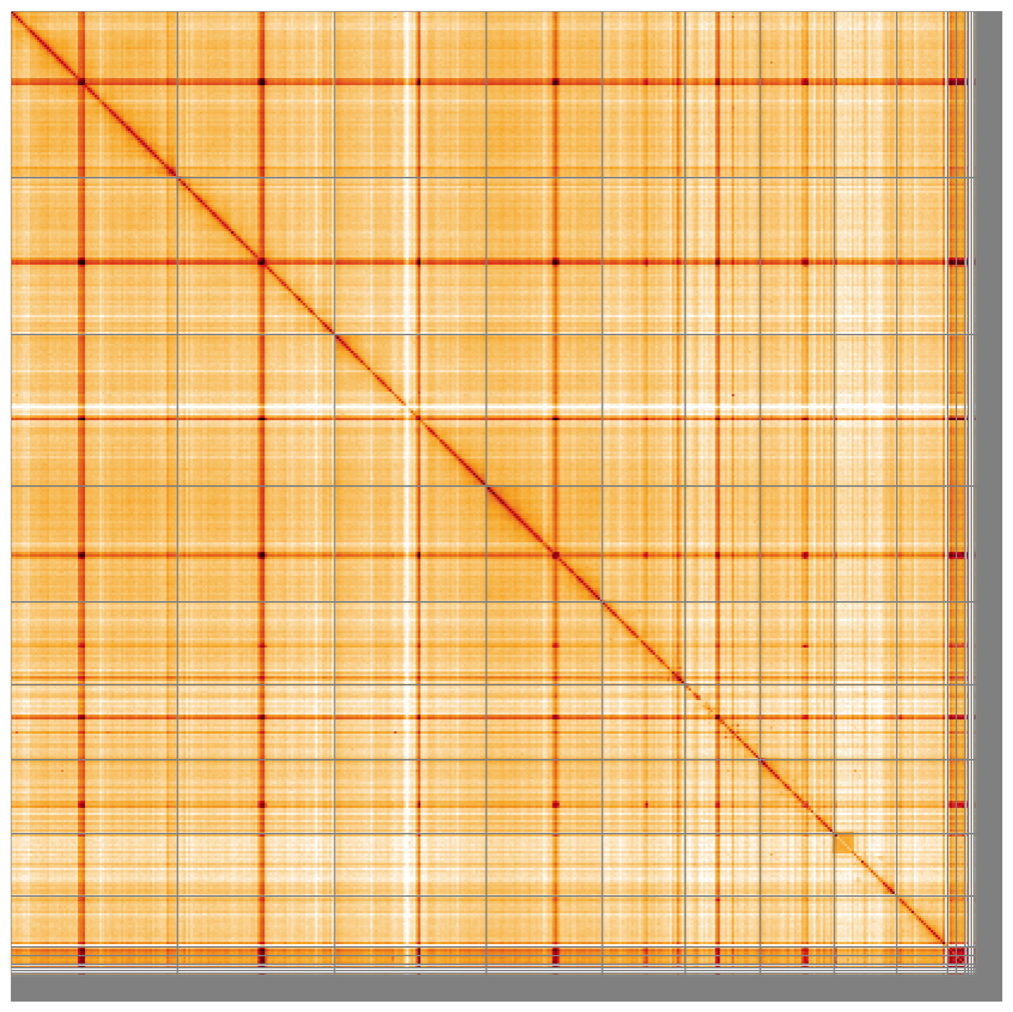
Genome assembly of *Bugula neritina* tzBugNeri2.1: Hi-C contact map of the tzBugNeri2.1 assembly, visualised using HiGlass. Chromosomes are shown in order of size from left to right and top to bottom. An interactive version of this figure may be viewed at https://genome-note-higlass.tol.sanger.ac.uk/l/?d=EsDQ2q5VTHeLSFoZVP3Pag.

Manual assembly curation corrected 17 missing joins or mis-joins and one haplotypic duplications, reducing the assembly length by 6.39% and the scaffold number by 50.08%. The final assembly has a total length of 216.00 Mb in 320 sequence scaffolds, with 110 gaps, and a scaffold N50 of 24.8 Mb ( Table 2). The snail plot in Figure 2 provides a summary of the assembly statistics, while the distribution of assembly scaffolds on GC proportion and coverage is shown in Figure 3. The cumulative assembly plot in Figure 4 shows curves for subsets of scaffolds assigned to different phyla. Most (92.7%) of the assembly sequence was assigned to 9 chromosomal-level scaffolds. Chromosome-scale scaffolds confirmed by the Hi-C data are named in order of size ( Figure 5; Table 3). A large number of repetitive sequences could not be uniquely assigned to a chromosome. While not fully phased, the assembly deposited is of one haplotype. Contigs corresponding to the second haplotype have also been deposited. The mitochondrial genome was also assembled and can be found as a contig within the multifasta file of the genome submission.

**Table 2.** Genome assembly data for *Bugula neritina*, tzBugNeri2.1.

Genome assembly		
Assembly name	tzBugNeri2.1	
Assembly accession	GCA_964035545.1	
*Accession of alternate haplotype*	*GCA_964035465.1*	
Span (Mb)	216.00	
Number of contigs	431	
Contig N50 length (Mb)	4.0	
Number of scaffolds	320	
Scaffold N50 length (Mb)	24.8	
Longest scaffold (Mb)	35.64	
Assembly metrics *		*Benchmark*
Consensus quality (QV)	53.7	*≥ 50*
*k*-mer completeness	99.99%	*≥ 95%*
BUSCO **	C:83.4%[S:82.7%,D:0.7%], F:7.5%,M:9.1%,n:954	*C ≥ 95%*
Percentage of assembly mapped to chromosomes	92.7%	*≥ 95%*
Sex chromosomes	None	*localised homologous pairs*
Organelles	Mitochondrial genome: 15.25 kb	*complete single alleles*
Genome annotation of assembly GCA_964035545.1 at Ensembl		
Number of protein-coding genes	20,264	
Number of non-coding genes	1,222	
Number of gene transcripts	33,722	

* Assembly metric benchmarks are adapted from column VGP-2020 of “Table 1: Proposed standards and metrics for defining genome assembly quality” from Rhie *et al.* (2021). ** BUSCO scores based on the metazoa_odb10 BUSCO set using version 5.4.3. C = complete [S = single copy, D = duplicated], F = fragmented, M = missing, n = number of orthologues in comparison. A full set of BUSCO scores is available at https://blobtoolkit.genomehubs.org/view/Bugula_neritina/dataset/GCA_964035545.1/busco.

**Table 3.** Chromosomal pseudomolecules in the genome assembly of *Bugula neritina*, tzBugNeri2.

INSDC accession	Name	Length (Mb)	GC%
OZ037765.1	1	35.64	34.0
OZ037766.1	2	33.58	33.5
OZ037767.1	3	32.42	34.5
OZ037768.1	4	24.8	34.5
OZ037769.1	5	17.72	34.5
OZ037770.1	6	16.05	35.0
OZ037771.1	7	15.81	34.5
OZ037772.1	8	13.35	35.0
OZ037773.1	9	10.88	34.5
OZ037774.1	MT	0.02	29.0

The estimated Quality Value (QV) of the final assembly is 53.7 with *k*-mer completeness of 99.99%, and the assembly has a BUSCO v5.4.3 completeness of 83.4% (single = 82.7%, duplicated = 0.7%), using the metazoa_odb10 reference set ( *n* = 954).

Metadata for specimens, BOLD barcode results, spectra estimates, sequencing runs, contaminants and pre-curation assembly statistics are given at https://links.tol.sanger.ac.uk/species/10212.

## Genome annotation report

The *Bugula neritina* genome assembly (GCA_964035545.1) was annotated at the European Bioinformatics Institute (EBI) on Ensembl Rapid Release. The resulting annotation includes 33,722 transcribed mRNAs from 20,264 protein-coding and 1,222 non-coding genes ( Table 2; https://rapid.ensembl.org/Bugula_neritina_GCA_964035545.1/Info/Index). The average transcript length is 7,394.61. There are 1.57 coding transcripts per gene and 8.23 exons per transcript.

## Methods

### Sample acquisition

An adult specimen of *Bugula neritina* (specimen ID MBA-220815-011A, ToLID tzBugNeri2) was collected from the Mayflower Marina, Plymouth, Devon, UK (latitude 50.36, longitude

–4.17) on 2022-08-15. The specimen was collected by Patrick Adkins, Freja Azzopardi and Rebekka Uhl and identified by John Bishop (all from the Marine Biological Association) and then preserved on dry ice.

The specimen used for Hi-C sequencing (specimen ID MBA-200715-001A, ToLID tzBugNeri1) was an adult specimen collected from Queen Anne's Battery Marina Visitors' Pontoon, Plymouth, Devon, UK (latitude 50.36, longitude –4.13) on 2020-07-15. The specimen was collected and identified by Patrick Adkins, John Bishop and Christine Wood, and preserved by liquid nitrogen.

The initial identification was verified by an additional DNA barcoding process according to the framework developed by Twyford *et al*. (2024). A small sample was dissected from the specimens and stored in ethanol, while the remaining parts of the specimen were shipped on dry ice to the Wellcome Sanger Institute (WSI). The tissue was lysed, the COI marker region was amplified by PCR, and amplicons were sequenced and compared to the BOLD database, confirming the species identification ( Crowley *et al.*, 2023). Following whole genome sequence generation, the relevant DNA barcode region was also used alongside the initial barcoding data for sample tracking at the WSI ( Twyford *et al.*, 2024). The standard operating procedures for Darwin Tree of Life barcoding have been deposited on protocols.io ( Beasley *et al.*, 2023).

### Nucleic acid extraction

The workflow for high molecular weight (HMW) DNA extraction at the Wellcome Sanger Institute (WSI) Tree of Life Core Laboratory includes a sequence of core procedures: sample preparation and homogenisation, DNA extraction, fragmentation and purification. Detailed protocols are available on protocols.io ( Denton *et al.*, 2023b). The tzBugNeri2 sample was weighed and dissected on dry ice ( Jay *et al.*, 2023). Tissue from the modular colony was homogenised using a PowerMasher II tissue disruptor ( Denton *et al.*, 2023a). HMW DNA was extracted in the WSI Scientific Operations core using the Automated MagAttract v2 protocol ( Oatley *et al.*, 2023). The DNA was sheared into an average fragment size of 12–20 kb in a Megaruptor 3 system ( Bates *et al.*, 2023). Sheared DNA was purified by solid-phase reversible immobilisation, using AMPure PB beads to eliminate shorter fragments and concentrate the DNA ( Strickland *et al.*, 2023). The concentration of the sheared and purified DNA was assessed using a Nanodrop spectrophotometer and Qubit Fluorometer using the Qubit dsDNA High Sensitivity Assay kit. Fragment size distribution was evaluated by running the sample on the FemtoPulse system.

### Sequencing

Pacific Biosciences HiFi circular consensus DNA sequencing libraries were constructed according to the manufacturers’ instructions. DNA sequencing was performed by the Scientific Operations core at the WSI on a Pacific Biosciences Sequel IIe instrument.

Hi-C data were generated from frozen tissue from tzBugNeri1 using the Arima-HiC v2 kit. In brief, frozen tissue (–80 °C) was fixed, and the DNA crosslinked using a TC buffer containing formaldehyde. The crosslinked DNA was then digested using a restriction enzyme master mix. The 5’-overhangs were then filled in and labelled with a biotinylated nucleotide and proximally ligated. The biotinylated DNA construct was fragmented to a fragment size of 400 to 600 bp using a Covaris E220 sonicator. The DNA was then enriched, barcoded, and amplified using the NEBNext Ultra II DNA Library Prep Kit, following manufacturers’ instructions. The Hi-C sequencing was performed using paired-end sequencing with a read length of 150 bp on an Illumina NovaSeq 6000 instrument.

### Genome assembly, curation and evaluation

***Assembly***

The HiFi reads were first assembled using Hifiasm ( Cheng *et al.*, 2021) with the --primary option. Haplotypic duplications were identified and removed using purge_dups ( Guan *et al.*, 2020). The Hi-C reads were mapped to the primary contigs using bwa-mem2 ( Vasimuddin *et al.*, 2019). The contigs were further scaffolded using the provided Hi-C data ( Rao *et al.*, 2014) in YaHS ( Zhou *et al.*, 2023) using the --break option. The scaffolded assemblies were evaluated using Gfastats ( Formenti *et al.*, 2022), BUSCO ( Manni *et al.*, 2021) and MERQURY.FK ( Rhie *et al.*, 2020).

The mitochondrial genome was assembled using MitoHiFi ( Uliano-Silva *et al.*, 2023), which runs MitoFinder ( Allio *et al.*, 2020) and uses these annotations to select the final mitochondrial contig and to ensure the general quality of the sequence.

***Assembly curation***

The assembly was decontaminated using the Assembly Screen for Cobionts and Contaminants (ASCC) pipeline (article in preparation). Flat files and maps used in curation were generated in TreeVal ( Pointon *et al.*, 2023). Manual curation was primarily conducted using PretextView ( Harry, 2022), with additional insights provided by JBrowse2 ( Diesh *et al.*, 2023) and HiGlass ( Kerpedjiev *et al.*, 2018). Scaffolds were visually inspected and corrected as described by Howe *et al*. (2021). Any identified contamination, missed joins, and mis-joins were corrected, and duplicate sequences were tagged and removed. The curation process is documented at https://gitlab.com/wtsi-grit/rapid-curation (article in preparation).

***Evaluation of the final assembly***

The final assembly was post-processed and evaluated using the three Nextflow ( Di Tommaso *et al.*, 2017) DSL2 pipelines: sanger-tol/readmapping ( Surana *et al.*, 2023a), sanger-tol/genomenote ( Surana *et al.*, 2023b), and sanger-tol/blobtoolkit ( Muffato *et al.*, 2024). The readmapping pipeline aligns the Hi-C reads using bwa-mem2 ( Vasimuddin *et al.*, 2019) and combines the alignment files with SAMtools ( Danecek *et al.*, 2021). The genomenote pipeline converts the Hi-C alignments into a contact map using BEDTools ( Quinlan & Hall, 2010) and the Cooler tool suite ( Abdennur & Mirny, 2020). The contact map is visualised in HiGlass ( Kerpedjiev *et al.*, 2018). This pipeline also generates assembly statistics using the NCBI datasets report ( Sayers *et al.*, 2024), computes *k*-mer completeness and QV consensus quality values with FastK and MERQURY.FK, and runs BUSCO ( Manni *et al.*, 2021) to assess completeness.

The blobtoolkit pipeline is a Nextflow port of the previous Snakemake Blobtoolkit pipeline ( Challis *et al.*, 2020). It aligns the PacBio reads in SAMtools and minimap2 ( Li, 2018) and generates coverage tracks for regions of fixed size. In parallel, it queries the GoaT database ( Challis *et al.*, 2023) to identify all matching BUSCO lineages to run BUSCO ( Manni *et al.*, 2021). For the three domain-level BUSCO lineages, the pipeline aligns the BUSCO genes to the UniProt Reference Proteomes database ( Bateman *et al.*, 2023) with DIAMOND ( Buchfink *et al.*, 2021) blastp. The genome is also split into chunks according to the density of the BUSCO genes from the closest taxonomic lineage, and each chunk is aligned to the UniProt Reference Proteomes database with DIAMOND blastx. Genome sequences without a hit are chunked with seqtk and aligned to the NT database with blastn ( Altschul *et al.*, 1990). The blobtools suite combines all these outputs into a blobdir for visualisation.

The genome assembly and evaluation pipelines were developed using nf-core tooling ( Ewels *et al.*, 2020) and MultiQC ( Ewels *et al.*, 2016), relying on the Conda package manager, the Bioconda initiative ( Grüning *et al.*, 2018), the Biocontainers infrastructure ( da Veiga Leprevost *et al.*, 2017), as well as the Docker ( Merkel, 2014) and Singularity ( Kurtzer *et al.*, 2017) containerisation solutions.

Table 4 contains a list of relevant software tool versions and sources.

**Table 4.** Software tools: versions and sources.

Software tool	Version	Source
BEDTools	2.30.0	https://github.com/arq5x/bedtools2
BLAST	2.14.0	ftp://ftp.ncbi.nlm.nih.gov/blast/executables/blast+/
BlobToolKit	4.3.7	https://github.com/blobtoolkit/blobtoolkit
BUSCO	5.4.3 and 5.5.0	https://gitlab.com/ezlab/busco
bwa-mem2	2.2.1	https://github.com/bwa-mem2/bwa-mem2
Cooler	0.8.11	https://github.com/open2c/cooler
DIAMOND	2.1.8	https://github.com/bbuchfink/diamond
fasta_windows	0.2.4	https://github.com/tolkit/fasta_windows
FastK	427104ea91c78c3b8b8b49f1a7d6bbeaa869ba1c	https://github.com/thegenemyers/FASTK
Gfastats	1.3.6	https://github.com/vgl-hub/gfastats
GoaT CLI	0.2.5	https://github.com/genomehubs/goat-cli
Hifiasm	0.19.8-r603	https://github.com/chhylp123/hifiasm
HiGlass	44086069ee7d4d3f6f3f0012569789ec138f42b84 aa44357826c0b6753eb28de	https://github.com/higlass/higlass
Merqury.FK	d00d98157618f4e8d1a9190026b19b471055b 22e	https://github.com/thegenemyers/MERQURY.FK
MitoHiFi	3	https://github.com/marcelauliano/MitoHiFi
MultiQC	1.14, 1.17, and 1.18	https://github.com/MultiQC/MultiQC
NCBI Datasets	15.12.0	https://github.com/ncbi/datasets
Nextflow	23.04.0-5857	https://github.com/nextflow-io/nextflow
PretextView	0.2	https://github.com/sanger-tol/PretextView
purge_dups	1.2.5	https://github.com/dfguan/purge_dups
samtools	1.16.1, 1.17, and 1.18	https://github.com/samtools/samtools
sanger-tol/ascc	-	https://github.com/sanger-tol/ascc
sanger-tol/ genomenote	1.1.1	https://github.com/sanger-tol/genomenote
sanger-tol/ readmapping	1.2.1	https://github.com/sanger-tol/readmapping
Seqtk	1.3	https://github.com/lh3/seqtk
Singularity	3.9.0	https://github.com/sylabs/singularity
TreeVal	1.0.0	https://github.com/sanger-tol/treeval
YaHS	1.2a.2	https://github.com/c-zhou/yahs

### Genome annotation

The Ensembl Genebuild annotation system ( Aken *et al.*, 2016) was used to generate annotation for the *Bugula neritina* assembly (GCA_964035545.1) in Ensembl Rapid Release at the EBI. Annotation was created primarily through alignment of transcriptomic data to the genome, with gap filling via protein-to-genome alignments of a select set of proteins from UniProt ( UniProt Consortium, 2019).

### Wellcome Sanger Institute – Legal and Governance

The materials that have contributed to this genome note have been supplied by a Darwin Tree of Life Partner. The submission of materials by a Darwin Tree of Life Partner is subject to the **‘Darwin Tree of Life Project Sampling Code of Practice’**, which can be found in full on the Darwin Tree of Life website here. By agreeing with and signing up to the Sampling Code of Practice, the Darwin Tree of Life Partner agrees they will meet the legal and ethical requirements and standards set out within this document in respect of all samples acquired for, and supplied to, the Darwin Tree of Life Project.

Further, the Wellcome Sanger Institute employs a process whereby due diligence is carried out proportionate to the nature of the materials themselves, and the circumstances under which they have been/are to be collected and provided for use. The purpose of this is to address and mitigate any potential legal and/or ethical implications of receipt and use of the materials as part of the research project, and to ensure that in doing so we align with best practice wherever possible. The overarching areas of consideration are:

•   Ethical review of provenance and sourcing of the material

•   Legality of collection, transfer and use (national and international)

Each transfer of samples is further undertaken according to a Research Collaboration Agreement or Material Transfer Agreement entered into by the Darwin Tree of Life Partner, Genome Research Limited (operating as the Wellcome Sanger Institute), and in some circumstances other Darwin Tree of Life collaborators.

## Data Availability

European Nucleotide Archive: *Bugula neritina* (ruby bryozoan). Accession number PRJEB66388; https://identifiers.org/ena.embl/PRJEB66388 ( Wellcome Sanger Institute, 2023). The genome sequence is released openly for reuse. The *Bugula neritina* genome sequencing initiative is part of the Darwin Tree of Life (DToL) project. All raw sequence data and the assembly have been deposited in INSDC databases. Raw data and assembly accession identifiers are reported in Table 1 and Table 2.

## Author information

Members of the Marine Biological Association Genome Acquisition Lab are listed here: https://doi.org/10.5281/zenodo.8382513.

Members of the Darwin Tree of Life Barcoding collective are listed here: https://doi.org/10.5281/zenodo.12158331

Members of the Wellcome Sanger Institute Tree of Life Management, Samples and Laboratory team are listed here: https://doi.org/10.5281/zenodo.12162482.

Members of Wellcome Sanger Institute Scientific Operations: Sequencing Operations are listed here: https://doi.org/10.5281/zenodo.12165051.

Members of the Wellcome Sanger Institute Tree of Life Core Informatics team are listed here: https://doi.org/10.5281/zenodo.12160324.

Members of the Tree of Life Core Informatics collective are listed here: https://doi.org/10.5281/zenodo.12205391.

Members of the Darwin Tree of Life Consortium are listed here: https://doi.org/10.5281/zenodo.4783558.
