# Supplementary material for: The genome sequence of the ruby bryozoan, Bugula neritina (Linnaeus, 1758)
Source: Wellcome Open Res. 2024 Sep 18;9:533. [Version 1] doi: 10.12688/wellcomeopenres.23056.1 (PMC11480708; doi:10.12688/wellcomeopenres.23056.1)
Supplement: Peer review history

# Reviewer report: Reviewer response for version 1

**DOI:** 10.21956/wellcomeopenres.25392.r103511

**Published:** 2024-10-15

**Authors:** Ferdinand Marlétaz

**Affiliations:** 1 Centre for Life’s Origin & Evolution, Department of Ecology, Evolution & Environment, University College London, London, UK

This reports described the sequencing and assembly of the bryozoan, *Bugula neritina* at chromsome scale. This is a welcome dataset for a group of organisms that is sometimes overlooked. Probably because of the type I genome architecture of bryozoan (see Hoencamp 2021), the HiC contact map is really difficult to read and does not bring much. As I usually comment, it would be great to have more details about HiC procedures. It is good to have an annotation, maybe specificy its Busco would be also interesting.

Are sufficient details of methods and materials provided to allow replication by others?

Yes

Is the rationale for creating the dataset(s) clearly described?

Yes

Are the datasets clearly presented in a useable and accessible format?

Yes

Are the protocols appropriate and is the work technically sound?

Yes

Reviewer Expertise:

genomics, zoology, phylogeny

I confirm that I have read this submission and believe that I have an appropriate level of expertise to confirm that it is of an acceptable scientific standard.

# Reviewer report: Reviewer response for version 1

**DOI:** 10.21956/wellcomeopenres.25392.r102194

**Published:** 2024-10-12

**Authors:** Emily Giles

**Affiliations:** 1 Universidad Austral de Chile, Valdivia, Los Ríos Region, Chile; 2 Biosecurity Group, Cawthron Institute, Nelson, Nelson, New Zealand

The authors report a genome assembly and annotation for *Bugula neritina*. They used PacBio, HiFi and HiC to generate a high quality assembly mapped to 9 pseudochromosomes and including 320 scaffolds. A primary assembly was generated using Hifiasm, haplotypic duplications were purged with purge_dups, then HiC reads were mapped to the primary assembly for scaffolding. The assembly was annotated using the Ensembl genebuild pipeline. In general, the manuscript is well written and clear, though the methods are not always detailed in the manuscript directly rather specification is supplied via links which requires the reader to search elsewhere for details.

**Overall, the results are robust, but I do have a few concerns/questions:**

1. Please provide a bit more information in the methods so that the reader does not have to go to your github page for details, i.e. curation process and blobtools methods.
2. Different specimens were used for the PacBio and HiC libraries. What was the rational for this? How would this affect the assembly? This should be explained.
3. The authors briefly describe their assembly curation and evaluation process, but it is not clear if contamination identified with blobtools was removed from the assembly, i.e does the assembly contain Mollusca, Brachiopoda, and Cnidaria?
4.  The assembly has decent BUSCO scores with the metazoan database, though complete BUSCOs are only at 84.8%. This could be due to the evolutionary history of the species/ composition of metazoan database skewed towards other lineages, but given that the annotation does not include ab initio prediction, many genes could have been missed in the annotation process. This should be stated somewhere. Furthermore, regarding the annotation pipeline, all cDNAs from ENA and RefSeq were used to predict cDNAs? Or a subset of transcriptomes was used? This is not clear.

Overall, these resources will be valuable for evolutionary studies in Bryozoa. It would be nice for non-Bryozoan experts to know how these resources contribute to currently available genomic resources for Bryozoan or other closely related taxa.

Are sufficient details of methods and materials provided to allow replication by others?

Partly

Is the rationale for creating the dataset(s) clearly described?

Partly

Are the datasets clearly presented in a useable and accessible format?

Yes

Are the protocols appropriate and is the work technically sound?

Yes

Reviewer Expertise:

Comparative genomics, genome assembly, genome annotation, marine genomics, transcriptomics, population genomics

I confirm that I have read this submission and believe that I have an appropriate level of expertise to confirm that it is of an acceptable scientific standard, however I have significant reservations, as outlined above.

# Reviewer report: Reviewer response for version 1

**DOI:** 10.21956/wellcomeopenres.25392.r100213

**Published:** 2024-10-09

**Authors:** Tim Yue Him Wong

**Affiliations:** 1 Shenzhen University, Shenzhen, China

This work reports the chromosome level assembly of B. neritina tzBugNeri2.1 which is based on 132x PacBio data and Arima2 Hi-C data. The assembly process included initial PacBio assembly generation , haplotig separation, and Hi-C based scaffolding. The mitochondrial genome was also assembled and reported. 

 While the authors spend a full paragraph in the introduction mentioning bryostatin and host-symbiont relationship, this work included no information about the symbiont genome or any potential bacterial genomic fragments (as a side product from Hi-C based scaffolding) were probably excluded as noise. 

 In my opinion, this work, in addition to host-symbiont interaction, is very important to the study of evodevo, in terms of understanding of the evolution of bryozoan, especially this work finally provided a comprehensive understanding on the list and structure of important developmental genes such as HOX, signaling pathway genes such as Wnt, BMP, Shh etc (Wong et al. 2014; Fuchs et al. 2011). This will eventually enable the accurate positioning of bryozoan (Ectoprocta) in the lophotrochozoan and provide novel insight into evolution of spiralian, such as the development of body axis. I think this can be further elaborate in the final paragraph of the introduction where you can include citation on B. neritina developmental gene studies.

 For the method part, I am concerned by the condition of the specimen, because the specimen in the photo in Figure 1 has brownish color while a healthy colony, should have dark purple color. I checked the method part and there were no content on the assessment of the DNA quality. Also, it said in the Genome annotation part that " Annotation was created primarily through alignment of transcriptomic data to the genome", yet none was mentioned on the origin of the transcritpome data (RNAseq? use existing RNAseq dataset in SRA?). If the authors used the specimen shown in Figure 1 to generate a transcriptome dataset, I think the gene annotation part may inevitably have bias toward a subset of genes relating to cell-death or genes relating to degradation.

Are sufficient details of methods and materials provided to allow replication by others?

Partly

Is the rationale for creating the dataset(s) clearly described?

Partly

Are the datasets clearly presented in a useable and accessible format?

Yes

Are the protocols appropriate and is the work technically sound?

Partly

Reviewer Expertise:

Single cell RNAseq, evo-devo, eco-devo, larval settlement and metamorphosis, IsoSeq, dark proteome

I confirm that I have read this submission and believe that I have an appropriate level of expertise to confirm that it is of an acceptable scientific standard, however I have significant reservations, as outlined above.

## References

[1] : Gene expression in bryozoan larvae suggest a fundamental importance of pre-patterned blastemic cells in the bryozoan life-cycle. *Evodevo* .2011;2(1) : 10.1186/2041-9139-2-13 13 10.1186/2041-9139-2-13 21645327 PMC3133996

[2] Transcriptome analysis elucidates key developmental components of bryozoan lophophore development;Wong, Y., Ryu, T., Seridi, L. et al. *Sci Rep* .(2014)4, 6534;10.1038/srep06534PMC419264225300304
